# The Therapeutical Properties of Koumys

**Published:** 1874-10-01

**Authors:** 


### Translations.

### THE THERAPEUTICAL PROPERTIES OF KOUMYS.

*Translated from Le Progres Medical, by Fred. J. Huse, M. D.*

MUCH has recently been said concerning koumys and, in a certain number of cases at least, it seems to have yielded very noticeable results. Whether these happy results will be confirmed by further observations, or whether they should be classed among those coincidences which are too often met with in the experimentation upon new remedies, remains to be seen; nevertheless it should not be forgotten that although koumys is as yet little known even in France, it has long been employed with success in Russia.

It is well known that koumys is the product of the fermentation of milk, and that the Coumans or Komanes, and also the Tartars, who prepare it from mare's milk, make it at certain seasons of the year their almost exclusive nourishment, while it also constitutes to the present day a large portion of the food of most of the nomadic hordes of Asia. Russian physicians having observed that the tribes who used this fermented milk were completely exempt from certain diseases of the respiratory organs, and especially from phthisis, attributed this precious immunity to koumys, and proceeded to prescribe its use to the tuberculous. The results were highly encouraging, and the employment of koumys in the treatment of phthisis was rapidly extended in Russia and the adjacent countries until one may now find quite a large number of establishments devoted to the manufacture of this substance. In France the first essays in the use of koumys are of very recent date. M. Schuepp made, in 1865, a study of this medicine, which he considered as "wonderful," but his endeavors, as well as those of M. Foussagrives, who also attributed a serious value to it, failed to attract serious attention. During the last few months, however, fresh experiments have been made in several hospitals, at the instigation of Dr. Landowski, and it is to the results of these that we desire to direct attention.

Koumys is a lactescent liquid, of whitish color, with a characteristic odor recalling that of whey, and a taste peculiarly sour and sharp, much resembling that of buttermilk; the carbonic acid which it contains renders it very lively, so much so that it has been termed champagne-milk.

Two principal varieties exist, depending upon the extent of the fermentation. Koumys No. 1, freshly prepared, contains in 1,000 parts: Lactic acid, 10 to 12; carbonic acid, 7 to 8; alcohol, 15 to 16. No. 2, longer put up: Lactic acid, 13 to 16; carbonic acid, 10 to 12; alcohol, 20 to 24. The carbonic acid and the alcohol vary in quantity in proportion with the percentage of sugar contained in the milk from which the koumys is prepared, hence they usually add a certain quantity of sugar of milk to equal parts of asses' milk and cows' milk, this being the mixture most highly recommended; mares' milk, moreover, is not employed, even in Russia, except at certain seasons of the year.

Concerning the results of the employment of koumys in the hospitals of Paris, we have only the article recently published in the *Bulletin de Therapeutique* by M. Urdy, and the first part of a description just begun by M. Landowski in the *Journal de Therapeutique.*

We find in the latter an interesting quotation from the writings of a monk who visited Tartary in 1233.

After describing the method of preparing the koumys or cosmos, he says: "Tunc gustant illum, et quando est temperate pungitivum, libunt. Curgit enim super linguam sicut vinum asperum bibitur, et postquam homo cessat bibere relinquit saporem super linguam lactis amigdalini, et multum reddit interiora hominis *Jucunda*, et etiam inebriat debilia capita; multam etiam provocat urinam." This description of the immediate effects of the use of koumys is thus confirmed by M. Urdy: "During the first few days there appear the symptoms of excitement that generally belong to all alcoholic beverages; the pulse gains in volume and force while at the same time it becomes a little less frequent, the face is flushed and the temperature raised. Persons of delicate constitutions sometimes exhibit a slight degree of tipsiness. The secretion of the urine becomes more abundant and its reaction slightly acid. These immediate effects are nearly always present, but they quickly pass away, and at the end of three or four days the toleration is generally found to be completely established. From that time the patient manifests unequivocal signs of improvement. There are three things which one might take as an indication of the salutary action of the koumys, and which have attracted our attention by their frequence: Sleep, though long unknown to the poor consumptive, again becomes possible, the appetite improves, and the weight of the individual increases. This increase amounted, in one case, to 13 pounds in twenty-seven days." This result is very fine, and we are almost tempted to say that it is too fine. However it may prove hereafter, the eight cases of phthisis reported by M. Urdy have experienced an improvement more or less marked, and, in certain cases they have succeeded in arresting vomiting.

Without going into such fanciful theories as those of Schuepp (that koumys was not only a sparkling, acidulous alcoholic milk, but above all a sort of yeast, an organization in the germ of the elements of the connective tissue of which the pathological retrogression constitutes the source and essential elements of tuberculosis), one can easily understand these results when he calls up the chemical constitution of koumys, and also comprehend the improvement observed by M. Urdy in a case of chronic albuminuria. It is, indeed, in these two conditions, phthisis and dropsy, that koumys has been administered, and although we repeat that the published results are still too scanty to authorize formal conclusions, it may yet be proper to admit that while koumys may not constitute a specific for phthisis, it is none the less a very valuable medicine. We ought to add that we saw very excellent results at the Hospital Lariboisiere in 1872 from the use of raw meat and alcohol, and the remembrance of these facts would lead us to believe all the good that is reported of koumys if we did not know that in such cases it is always necessary to make certain allowances.

RECENT CHEMICAL ANALYSIS OF KOUMYS.

| In 1,000 parts; | NO. 1. | NO. 2. |
|---|---|---|
| Casein, Albumen | 20 | 20 |
| Fatty matter | 8.5 | 8.5 |
| Lactine | 40 | 23 |
| Lactic acid | 7 | 8.5 |
| Carbonic acid | 6.5 | 1.3 |
| Alcohol | 2.2 | 30 |
| Salts (phosphates and chlorates) | 6 | 6 |

ACTION OF ERGOT ON THE INFANT.—Dr. E. R. Herschel, in the New York *Medical Record*, says a new-born infant, by accident, received half a teaspoonful (thirty drops) of Squibb's fluid extract of ergot. Efforts to vomit by the infant failed. In an hour it was seized with severe abdominal pains, recurring every fifteen minutes, and lasting about one minute. Slight tetanic contractions of the face and extremities were present, and four hours subsequently a diarrhœa set in. All the symptoms yielded to hot baths in twelve hours, though for two weeks there was a tendency to diarrhœa. This supports the opinion that ergot does not cause the death of the child during labor.

